# Colitis After SARS-CoV-2 Infection

**DOI:** 10.7759/cureus.26532

**Published:** 2022-07-03

**Authors:** Rajwinder Gill, Evan Siau

**Affiliations:** 1 Internal Medicine, Icahn School of Medicine at Mount Sinai Beth Israel, New York City, USA

**Keywords:** bebtelovimab, sequele of covid-19, viral colitis, infectious colitis, colitis, sars-cov-2 infection, sars-cov-2, covid-19

## Abstract

The severe acute respiratory syndrome coronavirus 2 (SARS-CoV-2) pandemic has affected our lives in a lot of different ways. We have observed a variety of clinical presentations in people infected with SARS-CoV-2 or coronavirus disease 2019 (COVID-19). Here, we present a case of COVID-19 who developed colitis ten days after an initial positive test for SARS-CoV-2.

## Introduction

The majority of COVID-19 cases present with pulmonary involvement, but it is known now that there are patients who present with gastrointestinal symptoms along with pulmonary involvement and some with gastrointestinal symptoms only [1]. The most common presenting symptoms of COVID-19 are fever and cough [2]. Gastrointestinal symptoms seen in COVID-19 are nausea, vomiting, diarrhea, abdominal pain, and anorexia. A minority of cases have been seen with acute abdomens like acute appendicitis, bowel ischemia, and acute pancreatitis [1].

## Case presentation

A 65-year-old male with a history of polysubstance use disorder, alcohol withdrawal, pancreatitis, bipolar disorder, and gout initially presented to the emergency department with a suicide attempt with intranasal heroin use two days prior to presentation. On review of systems, he was found to have nonbloody diarrhea associated with abdominal pain. He tested positive for the SARS-CoV-2 virus during this presentation but didn’t have respiratory symptoms. He received bebtelovimab and was discharged home after his symptoms resolved. Ten days later, he presented with new-onset diffuse abdominal pain associated with non-bloody diarrhea for two days. He was feeling nauseated and had two episodes of non-bloody, non-bilious vomiting. He denied cough, chest pain, shortness of breath, or leg swelling.

In the emergency department, a physical exam was noticeable for a temperature of 37.5 degrees Celsius, blood pressure of 128/92, heart rate of 98/minute, and oxygen saturation of 99% on room air. Abdominal exam was pertinent for soft, non-distended abdomen and tender to palpation in the left lower quadrant and right lower quadrant. He still tested positive for the SARS-CoV-2 virus. The initial laboratory workup was unremarkable and displayed in Table 1.

**Table 1:** Initial laboratory workup

Test	Results	Reference Range
White blood count (WBC)	9.0	4.5-11.00 k/µL
Hemoglobin	14	13.6-16.3 g/dL
Platelet	379	150-450 k/µL
Sodium	143	135-145 meq/L
Potassium	3.9	3.5-5.2 mmol/L
Chloride	107	96-108 mmol/L
Phosphorus	3.0	2.4-4.7 mg/dL
Magnesium	2.0	1.5-2.5 mg/dL
Creatinine	0.60	0.5-1.1 mg/dL
Blood urea nitrogen	22	6-23 mg/dL
Aspartate aminotransferase	33	1-35 U/L
Alanine aminotransferase	23	1-45 U/L
Alkaline phosphatase	77	38-126 U/L
C-reactive protein	2.24	<5.1 mg/L

**Figure 1:**
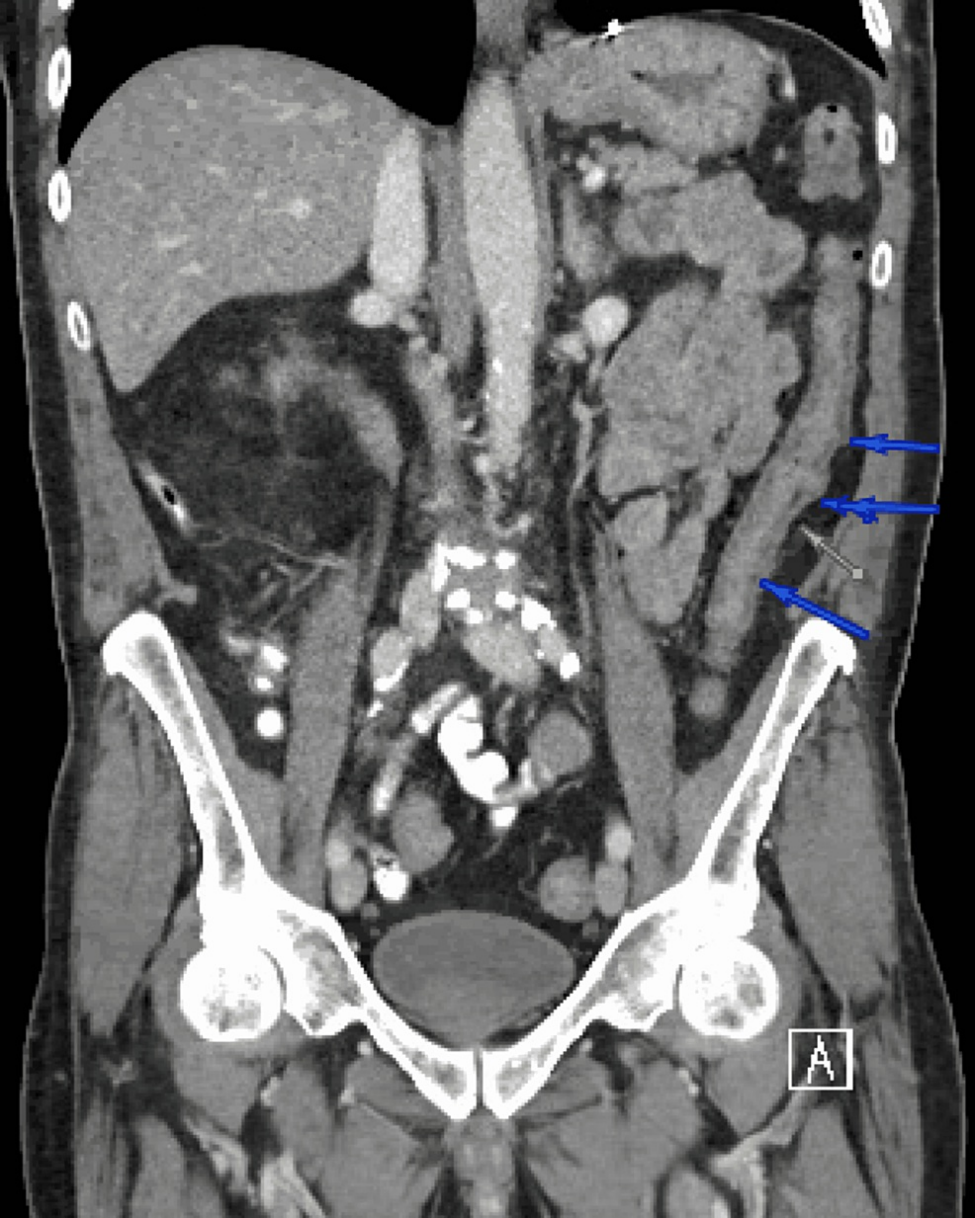
Computed tomography (CT) of abdomen and pelvis showing bowel wall thickening of descending colon

**Figure 2:**
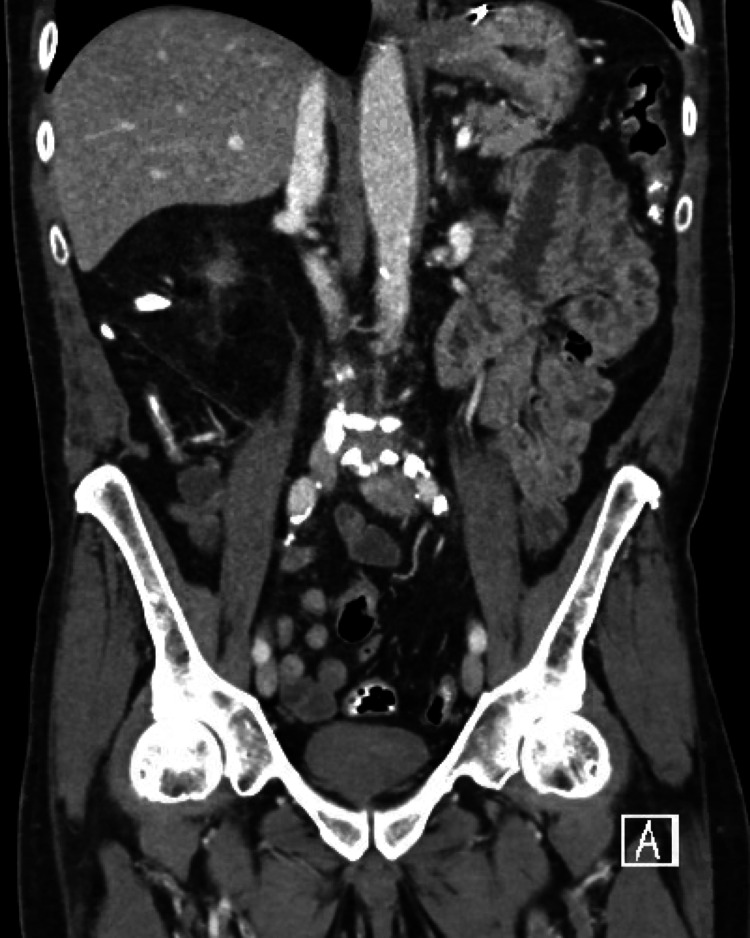
Previous CT scan without any evidence of colitis

Computed tomography (CT) of abdomen and pelvis was remarkable for left-sided colitis shown in Figure 1, this radiographic finding was not previously seen on the CT of abdomen and pelvis obtained on his initial admission 10 days prior as shown in Figure 2. We started him on ciprofloxacin and metronidazole as empirical treatment for bacterial infection, this regimen was stopped once gastrointestinal polymerase chain reaction testing (GI-PCR) was negative. Clostridium difficle testing was not done given no use of recent antibiotics. He had melena on day 2 of hospitalization but his hemoglobin level remained stable. His pain resolved on day 4 of hospitalization and he could tolerate oral intake with an improvement of overall clinical status and was discharged home.

## Discussion

We are seeing the different presentations and sequelae in patients with COVID-19. Some subset of people who had COVID-19 infection had long-lasting symptoms like persistent impairment of pulmonary function, reduction in diffusion capacity of lungs, decreased exercise tolerance, neuropsychiatric symptoms, chronic fatigue, and tachycardia. Some have postulated that gastrointestinal symptoms are seen in COVID-19 because gastrointestinal cells have high angiotensin convertase enzyme 2 (ACE2) expression. 15-20% of patients present with gastrointestinal symptoms. Some cases of acute COVID-19 have been found to have developed acute onset type I diabetes mellitus which has been explained by ACE2 expression on islet cells [3].

Post-acute SARS-CoV-2 syndrome is likely associated with persistent elevation of inflammatory markers like interferon-alpha, interferon-gamma, soluble T-cell immunoglobulin, and mucin domain-containing protein 3 (TIM3) [4]. As per the current literature, case of inflammatory bowel disease (IBD) after acute COVID-19 have been seen presenting with persistent diarrhea [5]. As per one study, 29% of the patients after acute SARS-CoV-2 infection had persistent gastrointestinal symptoms including nausea, vomiting, abdominal pain, and diarrhea [6]. Studies have shown a high amount of SARS-CoV-2 in gastrointestinal cells by detecting it in stool by nucleic acid amplification testing [7].

There has been a case of severe ulcerative colitis after COVID-19 which was fatal, in this case, SARS-CoV-2 infection was thought to trigger changes in immunomodulatory pathways [8]. Further studies on the gastrointestinal sequelae of COVID-19 are important to create strategies that would manage, treat, or prevent complications.

Our patient initially presented for suicidal attempt with intranasal heroin use two days prior to presentation, then he reported mild gastrointestinal symptoms with unremarkable vital signs, physical examination, and imaging including CT of his abdomen and pelvis was not revealing for any etiology; hence, raising the possibility that his initial presentation was consistent with early colitis associated with COVID-19 or they could be non-specific gastrointestinal symptoms observed in opiates withdrawal.

Additionally, this patient had received bebtelovimab before his presentation with gastrointestinal symptoms. Bebtelovimab is a monoclonal antibody that binds to the receptor-binding domain of spike protein present in the SARS-CoV-2 virus [9]. This raises the question if his subsequent presentation could be related to bebtelovimab administration. We are not aware of any current observation that has shown the association between bebtelovimab and colitis.

## Conclusions

Patients with COVID-19 who develop colitis may initially present with mild symptoms that can worsen and have delayed onset from the initial positive test for SARS-CoV-2. We need further studies to learn about the gastrointestinal sequelae of SARS-CoV-2 infection and further surveillance of potential adverse effects of monoclonal antibodies therapy for COVID-19.
